# Imaging features facilitate diagnosis of porto-sinusoidal vascular disorder

**DOI:** 10.1007/s00330-022-09132-4

**Published:** 2022-09-27

**Authors:** Katharina Lampichler, Georg Semmler, Katharina Wöran, Benedikt Simbrunner, Mathias Jachs, Lukas Hartl, David Josef Maria Bauer, Lorenz Balcar, Lukas Burghart, Michael Trauner, Dietmar Tamandl, Ahmed Ba-Ssalamah, Mattias Mandorfer, Thomas Reiberger, Bernhard Scheiner, Martina Scharitzer

**Affiliations:** 1 Department of Biomedical Imaging and Image-Guided Therapy, Medical University of Vienna, Vienna, Austria; 2 Division of Gastroenterology and Hepatology, Department of Internal Medicine III, Medical University of Vienna, Waehringer Guertel 18-20, 1090 Vienna, Austria; 3 Vienna Hepatic Hemodynamic Lab, Division of Gastroenterology and Hepatology, Department of Internal Medicine III, Medical University of Vienna, Vienna, Austria; 4 Rare Liver Disease (RALID) Center of the European Reference Network (ERN) RARE-LIVER, Medical University of Vienna, Vienna, Austria; 5 Clinical Institute of Pathology, Medical University of Vienna, Vienna, Austria; 6 Christian Doppler Laboratory for Portal Hypertension and Liver Fibrosis, Medical University of Vienna, Vienna, Austria; 7 Ludwig Boltzmann Institute for Rare and Undiagnosed Diseases, Vienna, Austria; 8 CeMM Research Center for Molecular Medicine of the Austrian Academy of Sciences, Vienna, Austria

**Keywords:** Liver cirrhosis, Portal vein, Multiparametric magnetic resonance imaging, Contrast agent

## Abstract

**Objectives:**

Porto-sinusoidal vascular disorder (PSVD) is a recently defined vascular liver disease. Since diagnosis remains challenging, we aimed to evaluate radiological features that are distinct between PSVD and cirrhosis.

**Methods:**

Clinical, laboratory, and radiological parameters (CT/MRI) of patients with histologically-confirmed PSVD vs. cirrhosis vs. non-cirrhotic parenchymal liver disease were retrospectively evaluated.

**Results:**

Sixty-three PSVD, 155 cirrhosis, and 41 non-cirrhotic patients were included. As compared to cirrhosis, PSVD patients were younger and had lower HVPG, liver stiffness, and MELD. Routine clinical and imaging findings indicative of portal hypertension were similarly common. Intrahepatic portal tract abnormalities (49% vs. 15%; *p* < 0.001), FNH-like lesions (30% vs. 1%; *p* < 0.001), and abnormal liver morphology defined as peripheral parenchymal atrophy and compensatory hypertrophy of central segments (32% vs. 7%; *p* < 0.001) were significantly more common in PSVD patients. Hypertrophy of segment I (70% vs. 84%; *p* = 0.019), atrophy of segment IV (24% vs. 47%; *p* = 0.001), and nodular liver surface (22% vs. 89%; *p* < 0.001) were more common in patients with cirrhosis. In patients with gadoxetic acid–enhanced MRI, we identified the distinct imaging feature of “periportal hyperintensity” in the hepatobiliary phase (HBP) in 42% of patients with PSVD (14/33) vs. 1% in cirrhosis (1/95) vs. 0% in non-cirrhotic controls (0/41); *p* < 0.001).

**Conclusions:**

Diagnosis of PSVD must be considered in younger patients presenting with clinical features of portal hypertension, portal tract abnormalities, and FNH-like lesions on CT/MRI. ‘Periportal hyperintensity’ in the HBP of gadoxetic acid–enhanced MRI was identified as a specific radiological feature of PSVD.

**Key Points:**

*• Cross-sectional imaging can provide essential information to identify patients with porto-sinusoidal vascular disorder (PSVD).*

*• Intrahepatic portal tract abnormalities, FNH-like lesions, and abnormal liver morphology are common in PSVD patients.*

*• Periportal hyperintensity on the hepatobiliary phase of gadoxetic acid–enhanced MRI seems to be specific for patients with PSVD.*

**Supplementary Information:**

The online version contains supplementary material available at 10.1007/s00330-022-09132-4.

## Introduction

Porto-sinusoidal vascular disorder (PSVD) is a novel vascular liver disease entity replacing and extending the previous term idiopathic non-cirrhotic portal hypertension (INCPH), due to relevant shortcomings of the latter definition. In contrast to INCPH, PSVD encompasses not only patients with clinical signs of portal hypertension (PH) in the absence of cirrhosis but also those with only specific histological features without PH that could not be assigned to a disease entity so far [1, 2]. Furthermore, the novel PSVD definition also accounts for the clinical notion that patients may have both, a vascular and parenchymal liver disease and does not exclude patients with portal vein thrombosis, in contrast to INCPH-definition [1].

From a clinical point of view, it is difficult to distinguish PSVD patients with clinical signs of PH from those with cirrhosis, which commonly leads to false or delayed diagnoses [3], even though certain features (e.g., lower liver stiffness measurement [LSM], lower hepatic venous pressure gradient [HVPG], and larger spleen-diameter in PSVD patients) might hint towards a vascular etiology of liver disease [4–6].

Cross-sectional imaging is commonly performed in patients with liver disease and may help to detect underlying liver pathology. Additionally, it provides important information for the identification and characterization of focal liver lesions and the assessment of PH severity (e.g., portosystemic collaterals) as well as prognosis [7–9]. In particular, multiparametric magnetic resonance imaging (MRI) with gadolinium-ethoxybenzyl-diethylenetriamine penta-acetic acid (Gd-EOB-DTPA) allows a detailed evaluation of liver disease [9–11]. While previous studies have reported on imaging findings in INCPH [12–14], only two studies have so far directly compared radiological findings in patients with PSVD to those of patients with cirrhosis [15, 16]. However, only 15 PSVD patients were evaluated using Gd-EOB-DTPA-enhanced MRI [15]. Thus, we further aimed to characterize radiological features in patients with PSVD, with a particular focus on Gd-EOB-DTPA-enhanced MRI.

## Methods

### Patients

Three patient cohorts treated at the Vienna General Hospital were evaluated for this retrospective study (Supplementary Figure 1). *Cohort I* included consecutive patients with PSVD treated between 01/2000 and 12/2020 with available contrast-enhanced cross-sectional liver imaging (computed tomography [CT] or MRI, *n* = 63). Specifically, all patients with Gd-EOB-DTPA-enhanced MRI were included since 2010. In brief, PSVD was diagnosed by retrospectively reviewing histological slides and clinical information in the absence of cirrhosis if either one specific clinical feature of PH, one specific histological feature of PSVD, or at least one unspecific clinical feature together with an unspecific histological feature were present, as according to the consensus definition [1]. Patients with cavernous transformation of the portal vein were excluded (n = 13) [1, 2]. *Cohort II* included patients with histologically proven cirrhosis participating in the prospective “VIenna CIrrhosis Study” (VICIS) between 01/2017 and 12/2020 in which cross-sectional liver imaging was available and who did not meet any exclusion criteria (*n* = 155). *Cohort III* included patients with non-cirrhotic parenchymal liver disease who underwent Gd-EOB-DTPA-enhanced MRI and served as additional controls to confirm the specificity of periportal hyperintensity for PSVD. In these patients, cirrhosis was ruled out histologically and none of these patients presented with features associated with PSVD (*n* = 41). For all three cohorts, exclusion criteria were insufficient imaging quality (e.g., breathing artefacts, poor imaging contrast, or those in which Gd-EOB-DTPA were not sufficiently taken up or excreted due to severely impaired liver function, insufficient liver biopsy specimens for a reliable diagnosis (i.e., those with a length of < 20 mm, including < 6 portal tracts and/or not considered to be adequate for histological evaluation by an expert pathologist) [1]. Further reasons for exclusion are presented in Supplementary Figure 1. Clinical and laboratory data at the time of imaging were extracted from patients’ medical records. Clinical characteristics of PSVD and cirrhosis patients as well as detailed histological results (PSVD patients) have in part been previously published [2, 17, 18].

### Imaging techniques

All patients underwent contrast-enhanced imaging assessment with CT and/or dynamic MRI of the liver using different vendors and scanner types given the long timespan of the study. Detailed information on scanner types, vendors, and contrast agents used can be found in the supplement. The minimal required set of MRI sequences included a coronal and axial T2-weighted half-Fourier single-shot turbo spin-echo sequence (HASTE, the latter with fat saturation) and three-dimensional T1 pre-contrast and dynamic contrast sequences in arterial, portal venous, and late phase including a hepatobiliary phase (HBP; 20 minutes after contrast injection), if applicable.

### Imaging analysis

Images were read in consensus by two board-certified radiologists K.L. and M.S. (7 and 15 years of experience in abdominal imaging). Evaluated imaging findings were as follows: Intra- und extrahepatic portal vein abnormalities including splanchnic vein thrombosis (SVT; partial or occlusive; acute or chronic), reduced caliber of intrahepatic portal vein branches, intrahepatic shunts, and intrahepatic collaterals. Intrahepatic shunts were defined as porto-venous or veno-venous shunts, which develop due to underlying liver damage, in contrast to intrahepatic collaterals, which develop due to portal vein thrombosis (PVT). The presence of FNH-like lesions was evaluated as previously defined [19]. For the assessment of liver morphology, the following imaging findings were evaluated: Caudate lobe hypertrophy (defined by the modified caudate-right lobe ratio > 0.65 [20]), atrophy of liver segment IV, or abnormal liver morphology which was defined as peripheral parenchymal atrophy and compensatory hypertrophy of central segments (segment I and the posterior segment IV) [21]. Perfusion inhomogeneity was defined as inhomogeneous contrast enhancement on the arterial or venous phase. Liver surface nodularity was evaluated visually (present or absent). Finally, the following signs of PH were evaluated: Extrahepatic portosystemic collaterals (present or absent), spleen size (splenomegaly was defined as ≥ 13 cm in maximum craniocaudal diameter), and ascites (present or absent).

The presence of periportal hyperintensity was evaluated in the HBP of Gd-EOB-DTPA-enhanced MRI and defined as follows: (1) continuous, linear periportal enhancement that is clearly visible; (2) presence within several liver segments (short periportal enhancement along only one portal vein segment was not included); (3) the sign was only assigned as present if no T1-weighted precontrast periportal hyperintensity was seen (i.e., false-positive periportal enhancement in the HBP).

### Measurement of HVPG and LSM, ethics, and statistical analyses

See supplementary materials.

## Results

### Patient characteristics

Important patient characteristics of PSVD and cirrhotic patients are summarized in Table 1. While the primary etiological factor identified in patients with PSVD was drug-induced (*n* = 29, 46%) followed by genetic (*n* = 12, 19%), most frequent etiologies in cirrhosis were alcoholic liver disease (*n* = 49, 32%), and viral hepatitis (*n* = 45, 29%). When comparing patients with PSVD to those with cirrhosis, patients with PSVD were younger (46.6 ± 16.5 vs. 56.4 ± 12.8, *p* < 0.001) and had a better liver function depicted as higher albumin (39.2 ± 5.7mg/dL vs. 36.4 ± 6.0 mg/dL, *p* = 0.001), lower UNOS-MELD score (9 ± 3 vs. 13 ± 6 points, *p* < 0.001), and a higher proportion of Child-Pugh (CPS)-class A compared to B and C (78% vs. 39% CPS-class A, 21% vs. 50% CPS-class B and 2% vs. 11% CPS-class C, *p* < 0.001). Importantly, the proportion of patients with a history of hepatic decompensation (46% vs. 54%, *p* = 0.27) and varices (59% vs. 54%, *p* = 0.54) was comparable between PSVD and patients with cirrhosis. Applying the proposed PSVD criteria for specific and unspecific clinical signs of PH [1] resulted in an even distribution of specific clinical signs (76% vs. 83%, *p* = 0.23) but a slightly lower proportion of unspecific clinical signs (84% vs. 94%, *p* = 0.017) in PSVD patients. HVPG was significantly lower in PSVD patients (8 [IQR: 4–11] mmHg), as compared to patients with cirrhosis (16 [IQR: 9–21] mmHg, *p* < 0.001), which was also true for LSM (9.0 [IQR: 6.5–12.2] kPa vs. 31.1 [IQR: 17.2–60.1] kPa, *p* < 0.001).

**Table 1:** Comparison of patient characteristics between PSVD and cirrhotic patients

	PSVD, *n* = 63	Cirrhosis, *n* = 155	*p* value
Age (years)	46.6 ± 16.5	56.4 ± 12.8	< 0.001
Female gender	23 (37%)	48 (31%)	0.429
BMI (kg/m^2^)	24.5 ± 4.7	26.8 ± 5.2	0.003
Etiology^1^			
ALD	-	49 (32%)	-
NAFLD		18 (12%)	
Viral		45 (29%)	
Cholestatic/autoimmune		16 (10%)	
Others		27 (17%)	
Drug-induced	29 (46%)^2^	-	
Genetic	12 (19%)^3^		
Immunological disorders	10 (16%)^4^		
Blood disease and prothrombotic conditions	3 (5%)		
Unclear	18 (29%)		
Albumin (mg/dL)	39.2 ± 5.7	36.4 ± 6.0	0.001
Platelet count (G/L)	109 (66–195)	111 (77–154)	0.752
CPS (points)	5 (5–6)	6 (5–8)	< 0.001
CPS A	49 (78%)	60 (39%)	< 0.001
CPS B	13 (21%)	78 (50%)	
CPS C	1 (2%)	17 (11%)	
UNOS-MELD (points)	9 ± 3	13 ± 6	< 0.001
Specific clinical signs of PH as according to PSVD consensus statement (1)	48 (76%)	129 (83%)	0.228
Unspecific clinical signs of PH as according to PSVD consensus statement (1)	53 (84%)	146 (94%)	0.017
History of decompensation	29 (46%)	84 (54%)	0.274
Ascites (clinically detectable)	15 (24%)	67 (43%)	0.007
Hepatic encephalopathy	1 (2%)	21 (18%)	0.001
Varices	37 (59%)	84 (54%)	0.541
Small	7 (19%)	42 (50%)	0.001
Large	30 (81%)	42 (50%)	
History of variceal bleeding	13 (21%)	18 (12%)	0.084
HVPG (mmHg)^5^	8 (4–11)	16 (9–21)	< 0.001
LSM (kPa)^6^	9.0 (6.5–12.2)	31.1 (17.2–60.1)	< 0.001

^1^Etiologies of PSVD were graded according to the presumed primary etiological trigger/factor. If 2 triggers existed, the patient was included in both groups; ^2^ Including 16 after azathioprine/thioguanine and 4 after chemotherapy; ^3^ Including 4 with cystic fibrosis and 1 with Turner’s syndrome; ^4^ Including 3 with HIV; ^5^ Available within < 90 days from imaging in 30 PSVD patients (48%) and 104 cirrhosis patients (67%); ^6^ Available within < 90 days from imaging in 29 PSVD patients (46%) and 94 cirrhosis patients (61%)

*Abbreviations: ALD* alcoholic liver disease; *NAFLD* non-alcoholic fatty liver disease; *BMI* bod mass index; *CPS* Child-Pugh core; *HVPG* hepatic venous pressure gradient; *LSM* liver stiffness measurement; *MELD* model for end-stage liver disease; *PH* portal hypertension; *PSVD* porto-sinusoidal vascular disorder

Baseline characteristics of controls with non-cirrhotic parenchymal liver disease can be found in Supplementary Table 1. In brief, predominant etiologies were non-alcoholic fatty liver disease (*n* = 28, 68%) and cholestatic/autoimmune liver diseases (*n* = 9, 22%).

### Differences in radiological features between PSVD and cirrhosis

**Fig. 1:**
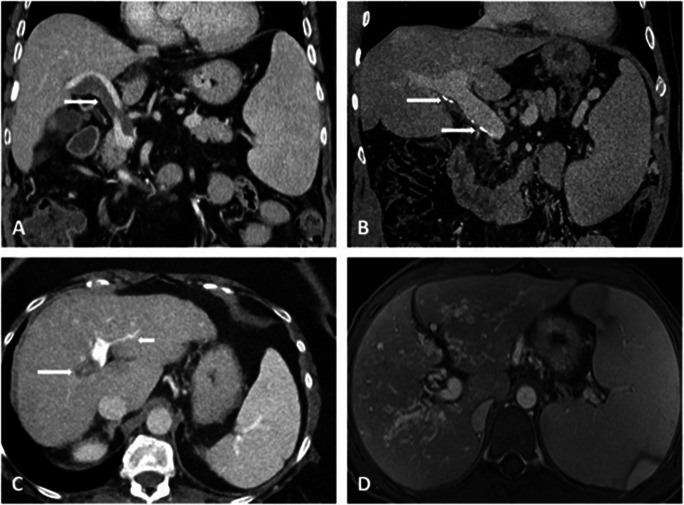
Different portal vein abnormalities in patients with PSVD: **A** 56-year-old male patient with acute, partial PVT of the main and right portal vein (white arrow). **B** 38-year-old male patient with chronic PVT and mural calcifications of the main portal vein (white arrows). **C** 62-year-old female patient with chronic, occlusive PVT of the right portal vein (white arrow) with small intrahepatic collaterals and reduced caliber of the branches of the left PV (short white arrow). **D** 37-year-old female patient with marked intrahepatic collaterals due to previous portal vein thrombosis with partial recanalization

**Fig. 2:**
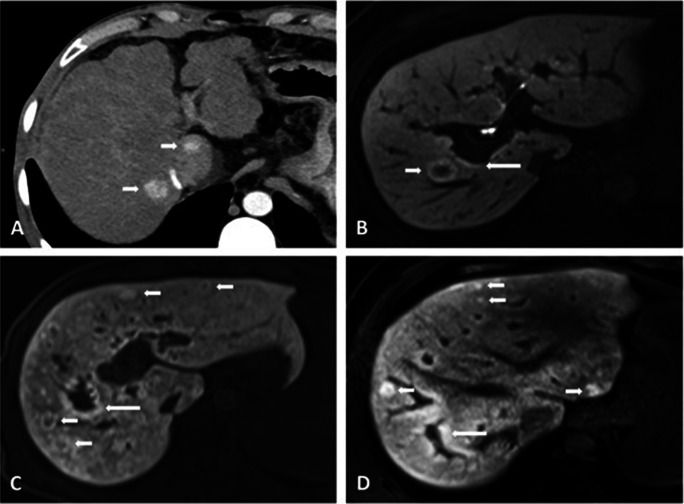
Different FNH-like lesions. **A** 34-year-old male patient with FNH-like lesions with strong arterial enhancement (short arrows) **B** 59-year-old male patient with a FNH-like lesion in segment VIII on HBP. Periportal hyperintensity is also present (large white arrow, B). **C** The same patient as in **D** with numerous FNH-like lesions on HBP (short white arrows). Periportal hyperintensity is also present (large white arrow). **D** 22-year-old male patient with several FNH-like lesions on HBP (short white arrows). Periportal hyperintensity is also present (large white arrows)

The median time between cross-sectional imaging and liver histology was 2.6 (IQR: 0.6–11.6) months. In line with clinical signs of PH, radiological signs of PH were equally prevalent among patients with PSVD and cirrhosis (Table 2). Specifically, the prevalence of portosystemic collaterals (76% vs. 81%, *p* = 0.40), splenomegaly (73% vs. 67%, *p* = 0.45), ascites (38% vs. 45%, *p* = 0.39), and SVT (19% vs. 11%, *p* = 0.11, Fig. 1A, B) was similar in PSVD and patients with cirrhosis. However, intrahepatic portal tract abnormalities were significantly more frequent in PSVD as compared to cirrhosis patients (49% vs. 15%, *p* < 0.001) and included the following findings: a reduced caliber of peripheral portal vein branches in 29% of PSVD (vs. 10% of patients with cirrhosis, *p* < 0.001, Fig. 1C), intrahepatic PVT in 16% (vs. 5%, *p* = 0.009, Fig. 2C), intrahepatic collaterals in 6% (vs. 0%, *p* = 0.006, Fig. 1D), and intrahepatic shunts in 19% (vs. 3%, *p* < 0.001), respectively. FNH-like lesions were identified in 19 patients with PSVD (30%, Fig. 2A–D) and in only two patients with cirrhosis (1%, *p* < 0.001).

**Table 2:** Comparison of radiological findings on CT/MRI between PSVD and cirrhotic patients

	PSVD, *n* = 63	Cirrhosis, *n* = 155	*p* value
MRI	36 (57%)	103 (66%)	0.125
Gd-EOB-DTPA-MRI	34 (54%)	102 (66%)	
CT	27 (43%)	52 (34%)	
Portosystemic collaterals	48 (76%)	126 (81%)	0.395
Spleen size (cm)^1^	15.2 ± 4.2	14.6 ± 3.5	0.332
Splenomegaly (≥ 13 cm)^1^	45 (73%)	103 (67%)	0.451
Splanchnic vein thrombosis (SVT)	12 (19%)	17 (11%)	0.111
Ascites on imaging	24 (38%)	69 (45%)	0.385
Any intrahepatic portal tract abnormalities	31 (49%)	23 (15%)	< 0.001
Reduced calibre of peripheral branches	18 (29%)	16 (10%)	< 0.001
Intrahepatic PVT	10 (16%)^4^	8 (5%)	0.009
Intrahepatic shunts	12 (19%)	4 (3%)	< 0.001
Intrahepatic collaterals	4 (6%)	0 (0%)	0.006
Perfusion disorder	21 (33%)	71 (46%)^5^	0.084
Hypertrophy of segment I	44 (70%)	130 (84%)	0.019
Atrophy of segment IV	15 (24%)	73 (47%)	0.001
Abnormal liver morphology^2^	20 (32%)	11 (7%)	< 0.001
Nodular surface	14 (22%)	138 (89%)	< 0.001
FNH-like lesions	19 (30%)	2 (1%)	< 0.001
Periportal hyperintensity^3^	14 (42%)	1 (1%)	< 0.001

^1^St.p. splenectomy in one PSVD patient and 2 cirrhotic patients; ^2^ Defined as peripheral parenchymal atrophy and compensatory hypertrophy of central segments and segment I; ^3^ Evaluable in patients with MRI scan of sufficient quality and well-preserved liver function to allow assessment of GA-excretion: *n* = 33 for PSVD (52%) and *n* = 95 for patients with cirrhosis (61%); ^4^ Signs of prior PVT were evident in 3 patients; ^5^ Perfusion could not be assessed in one patient due to respiratory artefacts

*Abbreviations*: *FNH* focal nodular hyperplasia; *Gd-EOB-DTPAGAGA* gadolinium-ethoxybenzyl-diethylenetriamine penta-acetic acid gadoxetic acid; *PSVD* porto-sinusoidal vascular disorder; *PVT* portal vein thrombosis; *SVT* splanchnic vein thrombosis

**Fig. 3:**
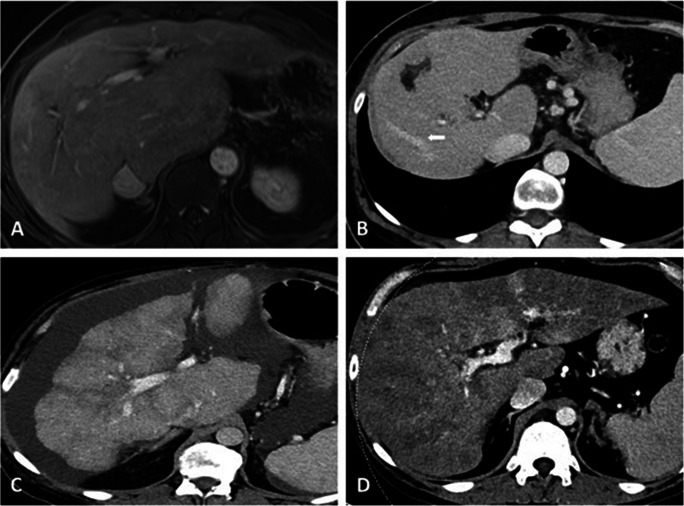
Different morphological changes of liver parenchyma in PSVD and cirrhosis. **A** 39-year-old female patient with PSVD and abnormal liver morphology with atrophy of the peripheral segments and central hypertrophy. **B** 50-year-old female patient with PSVD and a small, dysmorphic liver with atrophy of liver segments VI and VII (short white arrow marks the right liver vein) and central hypertrophy. **C** 51-year-old male patient with cirrhosis and hypertrophy of liver segment I as well as atrophy of liver segment IV and nodular liver surface. **D** 30-year-old patient with cirrhosis and marked perfusion inhomogeneities

PSVD patients more frequently presented with abnormal liver morphology (32% vs. 7%, *p* < 0.001, Fig. 3A, B). In contrast, atrophy of segment IV (24% vs. 47%, p = 0.001), nodular liver surface (22% vs. 89%, *p* < 0.001, Fig. 3C), and perfusion disorders (33% vs. 46%, *p* = 0.084, Fig. 3D) were more common in patients with cirrhosis. Subgroup analysis including only patients undergoing CT/MRI within one year from a liver biopsy can be found in the supplement.

### Imaging analysis—periportal hyperintensity on HBP

**Fig. 4:**
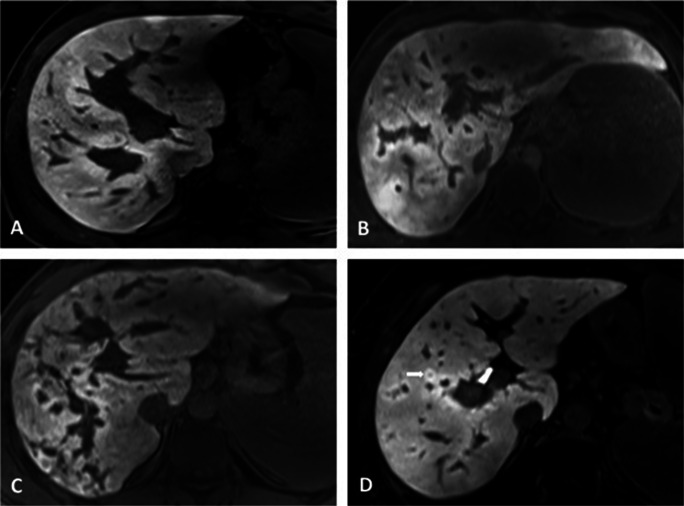
Periportal hyperintensity in patients with PSVD on HBP. **A** 22-year-old male patient with periportal hyperintensity along the left and right portal vein. Additional small FNH-like lesion in segment VIII (short white arrow). **B** 28-year-old male patient with periportal hyperintensity along the left and right portal vein. **C** 26-year-old male patient with periportal hyperintensity along the right portal vein. **D** 45-year-old female patient with periportal hyperintensity along the right portal vein. Small FNH-like lesion is also present (short arrow)

Gd-EOB-DTPA-enhanced MRI was available in 33 patients with PSVD (52%) and 95 patients with cirrhosis (61%). Periportal hyperintensity in the HBP was observed in 14 patients with PSVD (42%, Fig. 4A–D) but in only one patient with cirrhosis (1%, *p* < 0.001). Of note, periportal hyperintensity was not evident in any of the 41 patients with non-cirrhotic parenchymal liver disease. Additionally, none of the patients with periportal hyperintensity in the HBP had corresponding periportal edema on T2-weighted imaging.

### Differentiating PSVD from cirrhosis—the PSVD-radiology score

In order to provide a tool that may be useful for decision-making upon further validation, we incorporated the individual features with the highest discriminatory ability into a score assigning one point for “intrahepatic portal tract abnormalities,” “abnormal liver morphology,” “atrophy of segment IV,” “FNH-like lesions,” and “periportal hyperintensity” and deducting one point for “nodular liver surface.” Therefore, patients can achieve −2 to +4 points. Applying this approach, PSVD patients had a median score of 2 (IQR: 0–3) while patients with cirrhosis had −1 (IQR: −2–[−1], *p* < 0.001). Next, we calculated Youden’s index demonstrating that ≥ 1 point was the optimal cut-off for diagnosing PSVD (AUC: 0.913 [95%CI: 0.856–0.970]) with a sensitivity of 66.6%, a specificity of 98.9%, a positive likelihood-ratio of 60.5 and a negative likelihood-ratio of 0.34 for the diagnosis of PSVD. In total, 22/33 patients with PSVD and 94/95 patients with cirrhosis were correctly classified as having / not having PSVD (overall: 91%). Univariable and multivariable logistic regression analyses confirmed the importance of nodular liver surface and FNH-like lesions among those 5 characteristics (Supplementary Table 2). Probabilities for PSVD diagnosis using predictions from this multivariable logistic regression analysis are shown in a nomogram (Supplementary Figure 3).

## Discussion

In this study, we compared radiological features of PSVD with controls with cirrhosis or non-cirrhotic parenchymal liver diseases using cross-sectional imaging. We found that imaging features such as intrahepatic portal tract abnormalities, FNH-like lesions, and abnormal liver morphology should raise suspicion for PSVD[1]. Importantly, we could identify periportal hyperintensity on HBP as a specific feature in patients with PSVD. Finally, we developed a simple radiological score for the identification of PSVD which provided a high diagnostic accuracy.

The current study adds several aspects to increasing knowledge on radiological differences between patients with cirrhosis and PSVD patients [15, 16]. Valainathan and colleagues [16] recently provided a detailed characterization of radiomorphological changes specifically focusing on liver surface nodularity on CT imaging. Importantly, both the clinical characterization of included patients, but also imaging findings are strikingly similar to our cohorts, strengthening the validity of individual findings. Importantly, liver surface nodularity was confirmed in our study to be a central discriminator of PSVD vs. cirrhosis, however, quantification of liver surface nodularity as done by Valainathan and colleagues [16] requires a specific tool that is currently not widely available.

Furthermore, portal tract abnormalities on imaging were found in more than half of INCPH patients in another recent study by Kang and colleagues [15]. Interestingly, the same was true for our PSVD patients compared to only 15% of patients with cirrhosis making this another distinct feature in patients with PSVD. However, these 2 studies are harder to compare since Kang et al studied a selected subgroup of PSVD patients (applying the old INCPH definition [22], excluding all patients with a history of ‘hepatotoxic drug use’, including patients at a very advanced stage as histological proof was obtained at liver explantation in one-third of patients with PSVD) and only included 15 PSVD patients with Gd-EOB-DTPA-enhanced MRI [15]. Regarding aspects of “portal tract abnormalities,” we observed a considerable prevalence of intrahepatic collaterals due to occlusion of intrahepatic portal vein branches as well as a higher prevalence of intrahepatic porto-venous/veno-venous shunts next to previously described findings of narrowing of portal vein branches as well as occlusive or nonocclusive intrahepatic PVT [12].

Most importantly, we identified periportal hyperintensity on HBP as a very specific radiological feature in PSVD as this sign was observed in 42% of PSVD patients undergoing Gd-EOB-DTPA-enhanced MRI compared to only one patient with cirrhosis and not a single patient with non-cirrhotic parenchymal liver disease. While the pathophysiology of this finding is not well understood, reports in patients with INCPH as well as patients with a history of oxaliplatin-based chemotherapy hypothesized that regenerative changes in periportal hepatocytes leading to a relatively increased enhancement compared to the damaged background may be the underlying cause [23–25]. Kobayashi and colleagues [24] investigated the presence of periportal hyperintensity in a large sample of patients undergoing Gd-EOB-DTPA-enhanced MRI (*n* = 857 patients with various liver diseases of predominantly viral etiology and *n* = 256 healthy controls); however, they did not focus on vascular liver disease (aspects). They reported this feature in up to 3.2% with cirrhosis of viral etiology, 4 patients (5.1%) with alcoholic liver cirrhosis, 2 patients (12.5%) in primary biliary cirrhosis next to one patient (33.3%) with INCPH. We believe that periportal hyperintensity is indeed a feature of vascular liver disease/damage. The observation of this feature in a small proportion of patients with presumed cirrhosis could well be attributed to vascular changes in these patients. In our opinion, acknowledging that several liver diseases (parenchymal and vascular liver diseases) might be co-existing, is one of the major strengths of PSVD definition [1]. In line, the finding of periportal hyperintensity in one of our patients with parenchymal liver disease stimulates the hypothesis about the presence of vascular abnormalities in patients with (partially) regressive liver disease. This patient was diagnosed with histology-proven decompensated HCV cirrhosis and received direct-acting antiviral treatment five years prior to MRI. Even though LSM was significantly decreasing after etiological cure (last value of 5.4 kPa), clinically significant portal hypertension, varices, and mild ascites persisted. Additionally, the patient was diagnosed with intrahepatic PVT at the time of Gd-EOB-DTPA-enhanced MRI. Thus, although this patient was allocated to the cirrhosis group, a vascular component next to regressive parenchymal disease cannot be ruled out.

As a note of caution, several factors such as T1-hyperintensity on unenhanced images (Supplementary Figure 2B), patchy fatty infiltration, inhomogeneous or weak uptake in the HBP (Supplementary Figure 2C and 2D) and micronodular cirrhosis that may mimic periportal hyperintensity require careful evaluation. This also applies for the evaluation of other radiological features as intrahepatic portal veins may have a reduced caliber or may be missing due to advanced liver damage or drainage into large paraumbilical veins (Supplementary Figure 2A). Conversely, established imaging signs of cirrhosis (nodular liver surface, hypertrophy of segment I and atrophy of segment IV) were also observed in a substantial number of patients with PSVD and can therefore not reliably rule-out PSVD on cross-sectional imaging. Also, two other studies found that periportal hyperintensity could not only be observed in the HBP but also on T2-weighted images in some patients potentially corresponding to periportal edema representing active inflammation [26] or periportal fibrosis [27]. However, this could not be confirmed in our patient cohort.

Finally, we derived a simple score that could potentially assist to differentiate between PSVD and cirrhosis. Aiming at clinical applicability given the rarity of single characteristics in PSVD patients, the importance of each characteristic was considered equal for score development. However, multivariable regression underlined the importance of FNH-like lesions (PSVD) and nodular liver surface (cirrhosis). Thus, both, the PSVD-radiology score, but also the model estimating the probability of PSVD based on a nomogram require further validation.

An important strength of our study is the histological characterization of all controls: By only including histologically proven cirrhosis, we could confirm the specificity of radiological findings in patients with PSVD. Also, we included patients with histologically-confirmed non-cirrhotic parenchymal liver disease to rule out that periportal hyperintensity is a feature of non-cirrhotic liver disease. However, our study has some limitations: First, most of our PSVD patients had clinical signs of PH, thus, our results may not be generalized to a less advanced PSVD cohort, and further studies comparing PSVD patients without evidence for PH to those with non-cirrhotic parenchymal liver disease are strongly encouraged. However, we previously showed that clinical signs of PH are driving the development of liver-related outcomes, and therefore—in the absence of etiological therapies for PSVD—PSVD patients with signs of PH may have a more urgent need for diagnosis and treatment [2]. Second, matching patients with cirrhosis or non-cirrhotic controls according to liver disease severity (e.g., done by Kang and colleagues [15]) has some important drawbacks since PSVD patients usually have a well-preserved liver function [28], and therefore, matching patients by e.g. Child-Pugh-Score will lead to the comparison of compensated cirrhotic patients with impaired liver synthesis to decompensated PSVD patients. Therefore, every control cohort will be arbitrary to some degree. While other studies matched patients according to the presence of ascites in a 2:1 ratio [16], we chose to use consecutive patients from the VICIS study to gain a homogenous group, achieving a 2.5:1 matching with comparable specific signs of portal hypertension. Third, due to the retrospective design of our study, the time interval between liver biopsy and cross-sectional imaging was not standardized. Fourth, Gd-EOB-DTPA-enhanced MRI was only available in a subgroup of patients. Last, there is an ongoing debate on how to handle patients with cavernous transformation of the portal vein [29] as histological and radiological findings observed in these patients may be biased by the cavernous transformation, and may not be (anymore) attributed to the underlying disease entity. Thus, we deliberately chose to exclude patients with portal cavernoma from our analyses.

In conclusion, cross-sectional imaging provides important information for the non-invasive differentiation between PSVD, cirrhosis, and non-cirrhotic parenchymal liver disease. The presence of portal tract abnormalities, FNH-like lesions, the lack of liver surface nodularity, and segment IV atrophy should raise the suspicion of PSVD. Periportal hyperintensity on HBP was identified as a specific radiological feature of PSVD that could further guide the diagnostic workup. Validation of our findings and classification algorithms in multinational studies is warranted.

## A.Supplementary information

ESM 1 (DOCX 1060 kb)

## Abbreviations

ALD: Alcoholic liver disease
AUC: Area under the curve
BMI: Body mass index
CPS: Child-Pugh score
CT: Computed tomography
FNH: Focal nodular hyperplasia
Gd-EOB-DTPA: Gadolinium-ethoxybenzyl-diethylenetriamine penta-acetic acid
HBP: Hepatobiliary phase
HVPG: Hepatic venous pressure gradient
INCPH: Idiopathic non-cirrhotic portal hypertension
IQR: Interquartile range
LSM: Liver stiffness measurement
MELD: Model of end-stage liver disease
MRI: Magnetic resonance imaging
NAFLD: Non-alcoholic fatty liver disease
PH: Portal hypertension
PSVD: Porto-sinusoidal vascular disorder
PVT: Portal vein thrombosis
SVT: Splanchnic vein thrombosis

## Declarations

### Guarantor

The scientific guarantor of this publication is Thomas Reiberger.

### Statistics and biometry

Several authors have significant statistical expertise. Also, no complex statistical methods were necessary for this paper.

### Study subjects or cohorts overlap

In a prior study, we reported on the clinical characterization of patients with PSVD, being the first of its kind, as well as differences to patients meeting the former definition of INCPH [1]. This study included 91 patients with PSVD. Also, data on cirrhotic patients included in the prospective VICIS study (ClinicalTrials.gov identifier: NCT03541057) have been previously published (e.g., on 234 in the referred study) as this is a prospective registry study with consecutive sampling [2].

References

1. Wöran K, Semmler G, Jachs M et al (2020) Clinical course of porto-sinusoidal vascular disease is distinct from idiopathic noncirrhotic portal hypertension. Clin Gastroenterol Hepatol. 10.1016/j.cgh.2020.11.039

2. Simbrunner B, Semmler G, Stadlmann A et al (2020) Vitamin A levels reflect disease severity and portal hypertension in patients with cirrhosis. Hepatol Int 14:1093-1103

### Methodology

• retrospective

• Case control study

• performed at one institution
