# Supplementary material for: Imaging features facilitate diagnosis of porto-sinusoidal vascular disorder
Source: Eur Radiol. 2022 Sep 27;33(2):1422–32. doi: 10.1007/s00330-022-09132-4 (PMC9889423; doi:10.1007/s00330-022-09132-4)
Supplement: Supplementary file 1 — (DOCX 1060 kb) [file 330_2022_9132_MOESM1_ESM.docx]

**SUPPLEMENTARY METHODS**

**Imaging techniques**

Three different MRI scanners were used for *cohort I*: Siemens Magnetom Vision (Siemens Healthineers, Erlangen, Germany) using Gadobutrol (Gadovist®, Bayer HealthCare, Berlin, Germany) as well as Siemens Trio Tim and Siemens Prisma Fit (Siemens Healthineers, Erlangen, Germany) using gadolinium-ethoxybenzyl-diethylenetriamine penta-acetic acid (Gd-EOB-DTPA; Primovist®, Bayer HealthCare, Berlin, Germany) as contrast agent. Gadolinium-based contrast agents were administered adapted to body weight with a Gadolinium concentration between 150 and 300 mg/ml. For CT examinations, four different scanners were used in-house (Siemens volume zoom, Siemens Sensation Cardiac 64, Siemens Brilliance 64, and Siemens Somatom Drive) using either Jopamiro 300 or Iomeron 400 (Bracco, Milan, Italy) as contrast agent. Furthermore, scans from other institutions were performed using the following scanners: Philips Brilliance 16 (two patients), Siemens Definition AS+ and Toshiba Aquilion (one patient each). In *cohort II* (cirrhosis), CT scans were obtained using Siemens Brilliance 64 and Siemens Somatom Drive with intravenous administration of Iomeron 400. The different iodinated contrast agents were body weight adapted administered with an iodine concentration between 250 and 400 mg/ml. In *cohort III* (non-cirrhotic parenchymal liver disease), only patients with Gd-EOB-DTPA enhanced MRI (Siemens Trio Tim and Siemens Prisma Fit) were included.

**Measurement of hepatic venous pressure gradient and liver stiffness**

HVPG measurements were performed according to a standardized and published protocol using a 7 French balloon catheter (Pejcl Medizintechnik, Baden, Austria) [1]. Transjugular liver biopsy specimens were either obtained using an aspiration or core biopsy set, as previously described [1; 2]. LSM were performed using FibroScan® (Echosens, Paris, France) by experienced operators as previously described [3; 4]. The M and XL-probes were used according to the recommendations of the manufacturer. HVPG values and LSM were only considered if obtained within 90 days from CT/MRI scan.

**Ethics**

This study was approved by the ethics committee of the Medical University of Vienna (No. 1262/2017, 1928/2017, and 1889/2019). All patients in the prospective VICIS (ClinicalTrials.gov identifier: NCT03541057) and VALID (ClinicalTrials.gov identifier: NCT03541057) trial provided written informed consent. For the retrospective part of this study, the need for written informed consent was waived by the ethics committee.

**Statistical analyses**

Statistical analyses were performed using R 4.1.1 (R Core Team, R Foundation for Statistical Computing, Vienna, Austria). Continuous variables were reported as mean ± standard deviation or median (interquartile range [IQR]), and categorical variables were shown as numbers (n) and proportions (%) of patients. Comparisons of continuous variables were performed using Student *t*-test or Mann-Whitney-U-Test, as applicable. Comparisons of categorical variables were performed using Chi-squared or Fisher’s exact test. For development of a score to differentiate PSVD from cirrhosis based on cross-sectional imaging parameters, all parameters being significantly different (p<0.01) between the two cohorts were assigned one point (+1 for variables more prevalent in PSVD and -1 for variables more prevalent in cirrhosis). The area under the curve (AUC) and respective 95% confidence intervals of this score was calculated using the ‘pROC’-package. Youden’s cut-off was calculated to identify the cut-off with the highest discriminatory ability. Uni- and multivariable logistic regression analysis was performed to evaluate parameters significantly different between patients with PSVD and cirrhosis. A nomogram was created to display predicted probabilities of PSVD based on the multivariable regression model. A two-sided p-value ≤0.05 was considered statistically significant.

**SUPPLEMENTARY RESULTS**

**Sensitivity analysis in the subgroup of patients with cross-sectional imaging and liver histology within one year**

We performed a subgroup analysis only including patients in whom liver imaging and liver biopsy were performed within less than one year (n=53 [84%] for PSVD and n=112 [72.3%] for cirrhosis). Importantly, and as shown in Supplementary Tables 3 and 4, this approach did not change our results.

**SUPPLEMENTARY FIGURES**

**Supplementary Figure 1.** Patient flowchart.


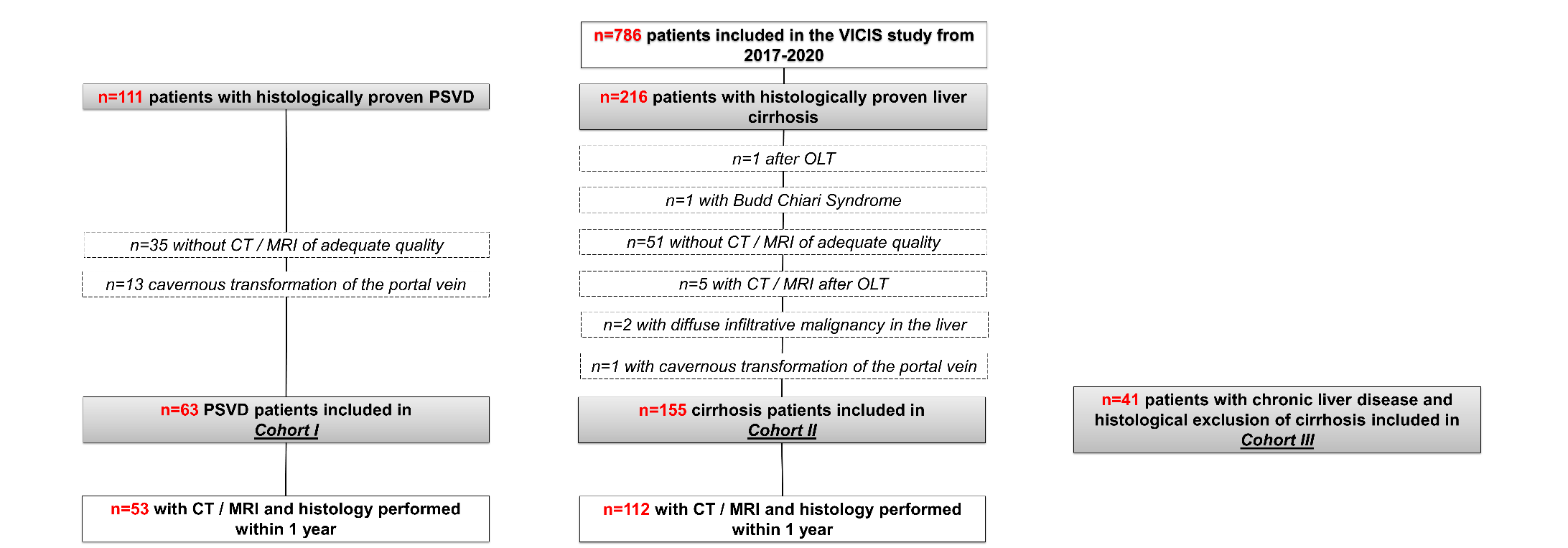


**Abbreviations:** OLT – orthotopic liver transplantation; PSVD – porto-sinusoidal vascular disorder

**Supplementary Figure 2.** Radiological pitfalls for differentiation of PSVD and cirrhosis **A** Large paraumbilical veins (short white arrow) in a 59-year old patient with cirrhosis caused a reduced caliber of intrahepatic portal veins and insufficient visuality of subsegmental portal veins, especially seen in the left liver lobe (large white arrows). **B** Periportal hyperintensity was mimicked in this 42-year old patient with cirrhosis as the hyperintensity is already seen on T1-weighted unenhanced imaging. **C** 33-year old patient with PSC. Advanced chronic liver disease resulted in patchy, partially reduced enhancement in the HBP, mimicking enhancement along the portal and hepatic veins. **D** 58-year old female patient with weak enhancement on HBP with faint periportal hyperintensity which should not be confused with true enhancement.


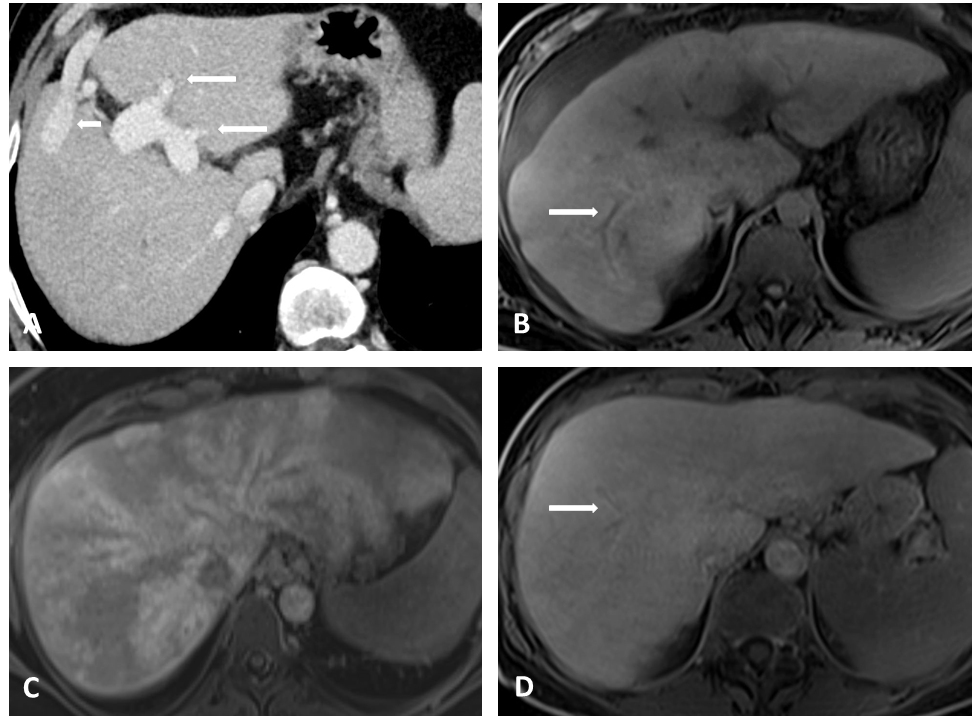


**Supplementary Figure 3.** Nomogram for the calculation of PSVD diagnosis probability based on predictions from multivariable logistic regression analysis incorporating all factors significantly different between PSVD vs. cirrhosis at a P-value of <0.01.


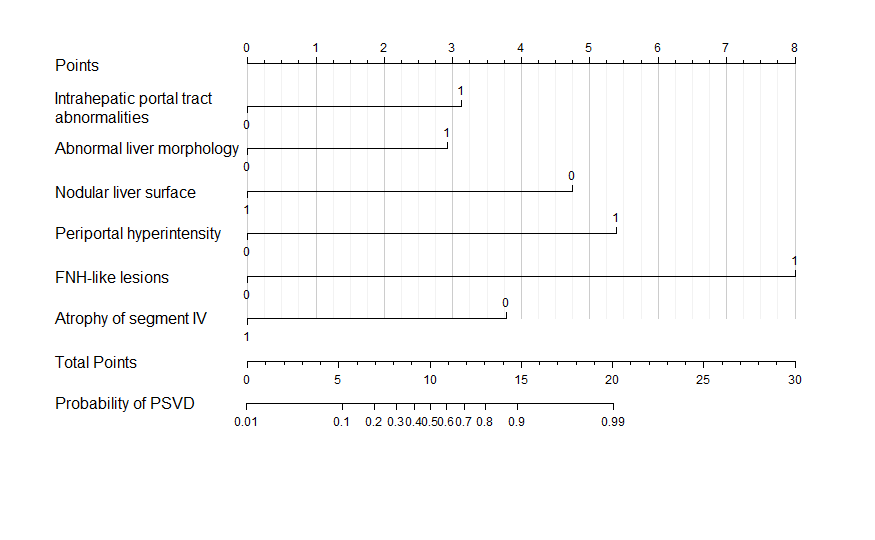


**SUPPLEMENTARY TABLES**

**Supplementary Table 1.** Patient characteristics and radiological findings on CT/MRI in non-cirrhotic chronic liver disease patients (Cohort III).

|  | **Non-cirrhotic,**  **n=41** |
| --- | --- |
| Age (years) | 48.6±13.4 |
| Female gender | 18 (44%) |
| BMI (kg/m²) | 27.5±5.5 |
| Etiology |  |
| ALD | 1 (2%) |
| NAFLD | 28 (68%) |
| Viral | 2 (5%) |
| Cholestatic/Autoimmune | 9 (22%) |
| Others | 1 (2%) |
| Specific clinical signs of PH as according to PSVD consensus statement [5] | 0 (0%) |
| Unspecific clinical signs of PH as according to PSVD consensus statement [5] | 9 (22.0%) |
| Ascites (on imaging) | 1 (2.4%) |
| Thrombocytopenia (<150G/L) | 4 (10%) |
| Splenomegaly (≥13cm) | 4 (10%) |
| History of hepatic decompensation | 0 (0%) |
| Ascites (clinically detectable) | 0 (0%) |
| Hepatic encephalopathy | 0 (0%) |
| LSM (kPa)^1^ | 7.8 (5.5-10.1) |

^1^ Available in 36 patients (87.8%).

**Abbreviations:** ALD – alcoholic liver disease; NAFLD – non-alcoholic fatty liver disease; BMI – bod mass index; LSM – liver stiffness measurement; PH – portal hypertension; PSVD – porto-sinusoidal vascular disorder

**Supplementary Table 2.** Univariable and multivariable logistic regression analyses investigating factors associated with PSVD.

|  | **Univariable OR (95%CI)** | ***P* value** | **Multivariable OR (95%CI)** | ***P* value** |
| --- | --- | --- | --- | --- |
| Any intrahepatic portal tract abnormalities | 10.64 (4.33-27.56) | **<0.001** | 4.17 (0.59-27.14) | 0.133 |
| Perfusion disorder | 0.59 (0.26-1.30) | 0.192 | - | - |
| Hypertrophy of segment I | 0.70 (0.26-1.99) | 0.478 | - | - |
| Atrophy of segment IV | 0.23 (0.07-0.59) | **0.005** | 0.18 (0.02-0.85) | 0.056 |
| Abnormal liver morphology | 9.00 (2.96-31.11) | **<0.001** | 3.80 (0.33-36.80) | 0.261 |
| Nodular surface | 0.06 (0.02-0.15) | **<0.001** | 0.11 (0.03-0.45) | **0.003** |
| FNH-like lesions | 43.76 (11.16-293.46) | **<0.001** | 11.79 (6.04-419.71) | **<0.001** |
| Periportal hyperintensity | 69.26 (12.79-1293.15) | **<0.001** | 38.79 (0.56-594.37) | 0.154 |

**Abbreviations:** FNH – focal nodular hyperplasia; OR – Odds ratio; PSVD – porto-sinusoidal vascular disorder; 95%CI – 95% confidence interval

**Supplementary Table 3.** Comparison of patient characteristics between PSVD and cirrhotic patients in the subgroup in which CT/MRI and liver histology were performed within 1 year.

|  | **PSVD,**  **n=53** | **Cirrhosis, n=112** | ***P* value** |
| --- | --- | --- | --- |
| Age (years) | 46.8±16.3 | 57.4±12.6 | **<0.001** |
| Female gender | 20 (38%) | 34 (30%) | 0.346 |
| BMI (kg/m²) | 24.6±4.9 | 26.8±5.1 | **0.009** |
| Albumin (mg/dL) | 39.1±5.8 | 35.2±5.7 | **<0.001** |
| Platelet count (G/L) | 107 (65-193) | 118 (79-161) | 0.673 |
| CPS (points) | 5 (5-6) | 7 (5-9) | **<0.001** |
| CPS A | 40 (75%) | 34 (30%) | **<0.001** |
| CPS B | 12 (23%) | 63 (56%) |  |
| CPS C | 1 (2%) | 15 (13%) |  |
| UNOS-MELD (points) | 9±3 | 14±7 | **<0.001** |
| Specific clinical signs of PH as according to PSVD consensus statement [5] | 39 (74%) | 93 (83%) | 0.156 |
| Unspecific clinical signs of PH as according to PSVD consensus statement [5] | 44 (83%) | 103 (92%) | 0.085 |
| History of decompensation | 24 (45%) | 65 (58%) | 0.125 |
| Ascites (clinically detectable) | 15 (28%) | 54 (48%) | **0.015** |
| Hepatic encephalopathy | 1 (2%) | 20 (25%) | **<0.001** |
| Varices | 32 (60%) | 56 (50%) | 0.168 |
| Small | 6 (19%) | 27 (48%) | **0.006** |
| Large | 26 (81%) | 29 (52%) |  |
| History of variceal bleeding | 10 (19%) | 10 (9%) | 0.068 |
| HVPG (mmHg)^1^ | 8 (4-11) | 17 (11-21) | **<0.001** |
| LSM (kPa)^2^ | 8.7 (6.1-12.1) | 35.3 (19.9-65.0) | **<0.001** |

^1^ Available within <90 days from imaging in 28 PSVD patients (53%) and 86 ACLD patients (77%); ^2^ Available within <90 days from imaging in 27 PSVD patients (51%) and 73 ACLD patients (65%).

**Abbreviations:** ALD – alcoholic liver disease; NAFLD – non-alcoholic fatty liver disease; BMI – bod mass index; CPS – Child Pugh Score; HVPG – hepatic venous pressure gradient; LSM – liver stiffness measurement; MELD – model for end-stage liver disease; PH – portal hypertension; PSVD – porto-sinusoidal vascular disorder

**Supplementary Table 4.** Comparison of radiological findings on CT/MRI between PSVD and cirrhotic patients in the subgroup in which CT/MRI and liver histology were performed within 1 year.

|  | **PSVD,**  **n=53** | **Cirrhosis,**  **n=112** | ***P* value** |
| --- | --- | --- | --- |
| Gd-EOB-DTPA-MRI | 28 (53%) | 69 (62%) | 0.297 |
| MRI | 2 (4%) | 1 (1%) |  |
| CT | 23 (43%) | 42 (38%) |  |
| Portosystemic collaterals | 40 (75%) | 92 (82%) | 0.317 |
| Spleen size (cm) | 15.0±4.2 | 14.3±3.5 | 0.280 |
| Splenomegaly (≥13cm) | 38 (73%) | 70 (64%)^4^ | 0.283 |
| Splanchnic vein thrombosis (SVT) | 11 (21%) | 11 (10%) | 0.054 |
| Ascites on imaging | 20 (38%) | 54 (48%) | 0.206 |
| Any intrahepatic portal tract abnormalities | 27 (51%) | 19 (17%) | **<0.001** |
| Reduced calibre of peripheral branches | 14 (26%) | 14 (13%) | **0.028** |
| Intrahepatic PVT | 9 (17%)^3^ | 5 (4%) | **0.007** |
| Intrahepatic shunts | 12 (23%) | 2 (2%) | **<0.001** |
| Intrahepatic collaterals | 4 (8%) | 0 (0%) | **0.009** |
| Perfusion disorders | 18 (34%) | 52 (46%) | 0.130 |
| Hypertrophy of segment I | 36 (68%) | 95 (85%) | **0.012** |
| Atrophy of segment IV | 14 (26%) | 48 (43%) | **0.042** |
| Abnormal liver morphology^1^ | 17 (32%) | 5 (4%) | **<0.001** |
| Nodular surface | 10 (19%) | 102 (91%) | **<0.001** |
| FNH-like lesions | 16 (30%) | 0 (0%) | **<0.001** |
| Periportal hyperintensity^2^ | 12 (43%) | 0 (0%) | **<0.001** |

^1^ Defined as peripheral parenchymal atrophy and compensatory hypertrophy of central segments and segment I; ^2^ Evaluable in patients with MRI scan and well preserved liver function to allow assessment of GA-excretion: n=27 for PSVD (51%) and n=63 for patients with cirrhosis (56%); ^3^ Signs for prior PVT were evident in 2 patients; ^4^ St.p. splenectomy in 2 patients;

**Abbreviations:** FNH – focal nodular hyperplasia; Gd-EOB-DTPAGA – gadolinium-ethoxybenzyl-diethylenetriamine penta-acetic acid; PSVD – porto-sinusoidal vascular disorder; PVT – portal vein thrombosis; SVT – splanchnic vein thrombosis

**References**

1 Reiberger T, Schwabl P, Trauner M, Peck-Radosavljevic M, Mandorfer M (2020) Measurement of the Hepatic Venous Pressure Gradient and Transjugular Liver Biopsy. JoVE. doi:10.3791/58819:e58819

2 Stift J, Semmler G, Walzel C et al (2019) Transjugular aspiration liver biopsy performed by hepatologists trained in HVPG measurements is safe and provides important diagnostic information. Dig Liver Dis 51:1144-1151

3 Reiberger T, Ferlitsch A, Payer BA et al (2012) Noninvasive screening for liver fibrosis and portal hypertension by transient elastography—a large single center experience. Wiener klinische Wochenschrift 124:395-402

4 Semmler G, Wöran K, Scheiner B et al (2020) Novel reliability criteria for controlled attenuation parameter assessments for non-invasive evaluation of hepatic steatosis. United European Gastroenterol J 8:321-331

5 De Gottardi A, Rautou PE, Schouten J et al (2019) Porto-sinusoidal vascular disease: proposal and description of a novel entity. Lancet Gastroenterol Hepatol 4:399-411
